# RTLola Cleared for Take-Off: Monitoring Autonomous Aircraft

**DOI:** 10.1007/978-3-030-53291-8_3

**Published:** 2020-06-16

**Authors:** Jan Baumeister, Bernd Finkbeiner, Sebastian Schirmer, Maximilian Schwenger, Christoph Torens

**Affiliations:** 8 Microsoft Research Lab, Redmond, WA USA; 9 University of Southern California, Los Angeles, CA USA; 10 Department of Computer Science, Saarland University, 66123 Saarbrücken, Germany; 11 German Aerospace Center (DLR), 38108 Braunschweig, Germany

**Keywords:** Runtime verification, Stream monitoring, FPGA, Autonomous aircraft

## Abstract

The autonomous control of unmanned aircraft is a highly safety-critical domain with great economic potential in a wide range of application areas, including logistics, agriculture, civil engineering, and disaster recovery. We report on the development of a dynamic monitoring framework for the DLR ARTIS (Autonomous Rotorcraft Testbed for Intelligent Systems) family of unmanned aircraft based on the formal specification language RTLola. RTLola is a stream-based specification language for real-time properties. An RTLola specification of hazardous situations and system failures is statically analyzed in terms of consistency and resource usage and then automatically translated into an FPGA-based monitor. Our approach leads to highly efficient, parallelized monitors with formal guarantees on the noninterference of the monitor with the normal operation of the autonomous system.

## Introduction

An unmanned aerial vehicle, commonly known as a drone, is an aircraft without a human pilot on board. While usually connected via radio transmissions to a base station on the ground, such aircraft are increasingly equipped with decision-making capabilities that allow them to autonomously carry out complex missions in applications such as transport, mapping and surveillance, or crop and irrigation monitoring. Despite the obvious safety-criticality of such systems, it is impossible to foresee all situations an autonomous aircraft might encounter and thus make a safety case purely by analyzing all of the potential behaviors in advance. A critical part of the safety engineering of a drone is therefore to carefully monitor the actual behavior during the flight, so that the health status of the system can be assessed and mitigation procedures (such as a return to the base station or an emergency landing) can be initiated when needed.

In this paper, we report on the development of a dynamic monitoring framework for the DLR ARTIS (Autonomous Rotorcraft Testbed for Intelligent Systems) family of aircraft based on the formal specification language RTLola. The development of a monitoring framework for an autonomous aircraft differs significantly from a monitoring framework in a more standard setting, such as network monitoring. A key consideration is that while the specification language needs to be highly *expressive*, the monitor must operate within strictly limited resources, and the monitor itself needs to be highly *reliable*: any interference with the normal operation of the aircraft could have fatal consequences.

A high level of expressiveness is necessary because the assessment of the health status requires complex analyses, including a cross-validation of different sensor modules such as the agreement between the GPS module and the accelerometer. This is necessary in order to discover a deterioration of a sensor module. At the same time, the expressiveness and the precision of the monitor must be balanced against the available computing resources. The reliability requirement goes beyond pure correctness and robustness of the execution. Most importantly, reliability requires that the peak resource consumption of the monitor in terms of energy, time, and space needs to be known ahead of time. This means that it must be possible to compute these resource requirements statically based on an analysis of the specification. The determination whether the drone is equipped with sufficient hardware can then be made before the flight, and the occurrence of dynamic failures such as running out of memory or sudden drops in voltage can be ruled out. Finally, the collection of the data from the on-board architecture is a non-trivial problem: While the monitor needs access to almost the complete system state, the data needs to be retrieved non-intrusively such that it does not interfere with the normal system operation.

Our monitoring approach is based on the formal stream specification language RTLola [11]. In an RTLola specification, input streams that collect data from sensors, networks, etc., are filtered and combined into output streams that contain data aggregated from multiple sources and over multiple points in time such as over sliding windows of some real-time length. Trigger conditions over these output streams then identify critical situations. An RTLola specification is translated into a monitor defined in a hardware description language and subsequently realized on an FPGA. Before deployment, the specification is checked for consistency and the minimal requirements on the FPGA are computed. The hardware monitor is then placed in a central position where as much sensor data as possible can be collected; during the execution, it then extracts the relevant information. In addition to requiring no physical changes to the system architecture, this integration incurs no further traffic on the bus.

Our experience has been extremely positive: Our approach leads to highly efficient, parallelized monitors with formal guarantees on the non-interference of the monitor with the normal operation of the autonomous system. The monitor is able to detect violations to complex specifications without intruding into the system execution, and operates within narrow resource constraints. RTLola is cleared for take-off.

### Related Work

Stream-based monitoring approaches focus on an expressive specification language while handling non-binary data. Its roots lie in synchronous, declarative stream processing languages like Lustre [13] and Lola [9]. The *Copilot* framework [19] features a declarative data-flow language from which constant space and constant time C monitors are generated; these guarantees enable usage on an embedded device. Rather than focusing on data-flow, the family of Lola-languages puts an emphasis on statistical measures and has successfully been used to monitor synchronous, discrete time properties of autonomous aircraft [1, 23]. In contrast to that, RTLola [12, 22] supports real-time capabilities and efficient aggregation of data occurring with arbitrary frequency, while forgoing parametrization for efficiency [11]. RTLola can also be compiled to VHDL and subsequently realized on an FPGA [8].

Apart from stream-based monitoring, there is a rich body of monitoring based on real-time temporal logics [2, 10, 14–16, 20] such as Signal Temporal Logic (STL) [17]. Such languages are a concise way to describe temporal behaviors with the shortcoming that they are usually limited to qualitative statements, i.e. boolean verdicts. This limitation was addressed for STL [10] by introducing a quantitative semantics indicating the robustness of a satisfaction. To specify continuous signal patterns, specification languages based on regular expressions can be beneficial, e.g. Signal Regular Expressions (SRE) [5]. The R2U2 tool [18] stands out in particular as it successfully brought a logic closely related to STL onto unmanned aerial systems as an external hardware implementation.

## Setup

The Autonomous Rotorcraft Testbed for Intelligent Systems (ARTIS) is a platform used by the Institute of Flight Systems of the German Aerospace Center (DLR) to conduct research on autonomous flight. It consists of a set of unmanned helicopters and fixed-wing aircraft of different sizes which can be used to develop new techniques and evaluate them under real-world conditions.

**Fig. 1.**
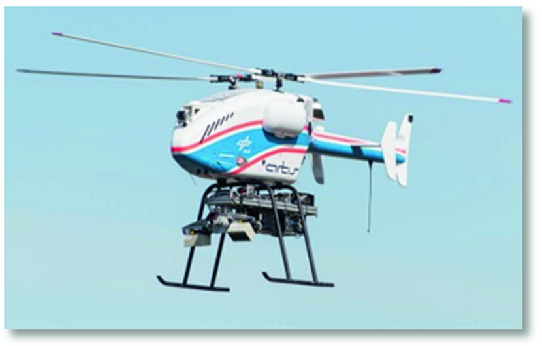
DLR’s autonomous superARTIS equipped with optical navigation.

The case study presented in this paper revolves around the superARTIS, a large helicopter with a maximum payload of 85 kg, depicted in Fig. 1. The high payload capabilities allow the aircraft to carry multiple sensor systems, computational resources, and data links. This extensive range of avionic equipment plays an important role in improving the situational awareness of the aircraft [3] during the flight. It facilitates safe autonomous research missions which include flying in urban or maritime areas, alone or with other aircraft. Before an actual flight test, software- and hardware-in-the-loop simulations, as well as real-time logfile replays strengthen confidence in the developed technology.

### Mission

One field of application for unmanned aerial vehicles (UAVs) is reconnaissance missions. In such missions, the aircraft is expected to operate within a fixed area in which it can cause no harm. The polygonal boundary of this area is called a geo-fence. As soon as the vehicle passes the geo-fence, mitigation procedures need to be initiated to ensure that the aircraft does not stray further away from the safe area.

**Fig. 2.**
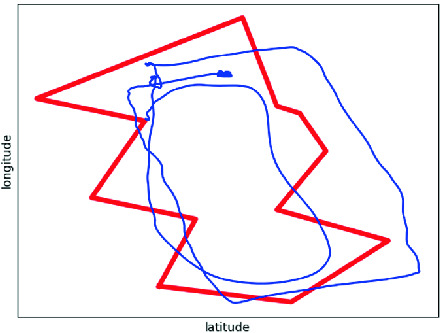
Reconnaissance mission for a UAV. The thin blue line represents its trajectory, the thick red line a geo-fence.

The case study presented in this paper features a reconnaissance mission. Figure 2 shows the flight path (blue line) within a geo-fence (red line). Evidently, the aircraft violates the fence several times temporarily. A reason for this can be flawed position estimation: An aircraft estimates its position based on several factors such as landmarks detected optically or GPS sensor readings. In the latter case, GPS satellites send position and time information to earth. The GPS module uses this data to compute the aircraft’s absolute position with trilateration. However, signal reflection or a low number of GPS satellites in range can result in imprecisions in the position approximation. If the aircraft is continuously exposed to imprecise position updates, the error adds up and results in a strong deviation from the expected flight path.

**Fig. 3.**
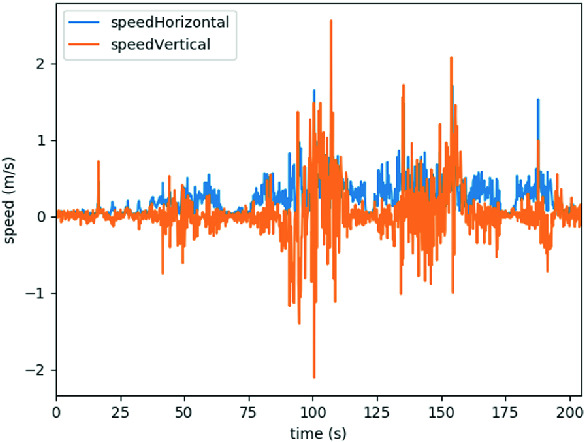
Line plot of the horizontal and vertical speed calculated by a GPS receiver.

The impact of this effect can be seen in Fig. 3. It shows the velocity of a ground-borne aircraft in an enclosed backyard according to its GPS module.^1^ During the reported period of time, the aircraft was pushed across the backyard by hand. While the expected graph is a smooth curve, the actual measurements show an erratic curve with errors of up to $\pm 1.5\,\text {ms}^{-1}$, which can be mainly attributed to signals being reflected on the enclosure. The strictly positive trend of the horizontal velocity can explain strong deviations from the desired flight path seen in Fig. 3.

A counter-measure to these imprecisions is the cross-validation of several redundant sensors. As an example, rather than just relying on the velocity reported by a GPS module, its measured velocity can be compared to the integrated output of an accelerometer. When the values deviate strongly, the values can be classified as less reliable than when both sensors agree.

### Non-Intrusive Instrumentation

When integrating the monitor into an existing system, the system architecture usually cannot be altered drastically. Moreover, the monitor should not interfere with the regular execution of the system, e.g. by requiring the controller to send explicit messages to it. Such a requirement could offset the timing behavior and thus have a negative impact on the overall performance of the system.

**Fig. 4.**
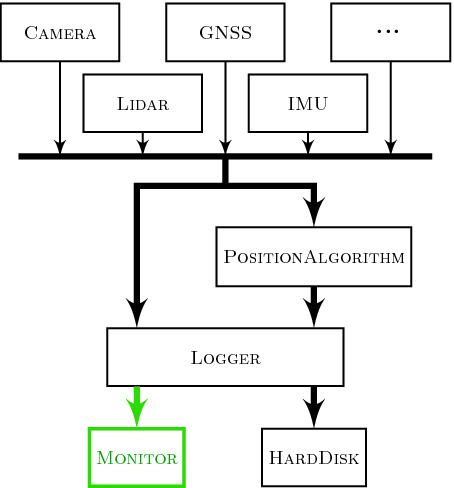
Overview of data flow in system architecture.

The issue can be circumvented by placing the monitor at a point where it can access all data necessary for the monitoring process non-intrusively. In the case of the superARTIS, the logger interface provides such a place as it compiled the data of all position-related sensors as well as the output of the position estimation [3, 4]. Figure 4 outlines the relevant data lines of the aircraft. Sensors were polled with fixed frequencies of up to 100 Hz. The schematic shows that the logger explicitly sends data to the monitor. This is not a strict requirement of the monitor as it could be connected to the data buses leading to the logger and passively read incoming data packets. However, in the present setting, the logger did not run at full capacity. Thus sending information to the monitor came at no relevant cost while requiring few hardware changes to the bus layout.

In turn, the monitor provides feedback regarding violations of the specification. Here, we distinguish between different timing behaviors of triggers. The monitor evaluates event-based triggers whenever the system passes new events to the monitor and immediately replies with the results. For periodic triggers, i.e. , those annotated with an evaluation frequency, the evaluation is decoupled from the communication between monitor and system. Thus, the monitor needs to wait until it receives another event until reporting the verdict. This incurs a short delay between detection and report.

### StreamLAB

StreamLAB^2^ [11] is a monitoring framework revolving around the stream-based specification language RTLola. It emphasizes on analyses conducted before deployment of the monitor. This increases the confidence in a successful execution by providing information to aid the specifier. To this end, it detects inconsistencies in the specification such as type errors, e.g. an lossy conversion of a floating point number to an integer, or timing errors, e.g. accessing values that might not exist. Further, it provides two execution modes: an interpreter and an FPGA compilation. The interpreter allows the specifier to validate their specification. For this, it requires a *trace*, i.e. a series of data that is expected to occur during an execution of the system. It then checks whether a trace complies with the specification and reports the points in time when specified bounds are violated. After successfully validating the specification, it can be compiled into VHDL code. Yet again, the compiled code can be analyzed with respect to the space and power consumption. This information allows for evaluating whether the available hardware suffices for running the RTLola monitor.

An RTLola specification consists of input and output streams, as well as trigger conditions. *Input* streams describe data the system produces asynchronously and provides to the monitor. *Output* streams use this data to assess the health state of the system e.g. by computing statistical information. *Trigger* conditions distinguish desired and undesired behavior. A violation of the condition issues an alarm to the system.

The following specification declares a floating point input stream  representing sensor readings of an altimeter. The output stream  computes the average value of the  stream over two minutes. The aggregation is a sliding window computed once per second, as indicated with the  annotation.^3^ The stream  computes the difference between the average and the current height. A strong deviation of these values constitutes a suspicious jump in sensor readings, which might indicate a faulty sensor or an unexpected loss or gain in height. In this case, the trigger in the specification issues a warning to the system, which can initiate mitigation measures. 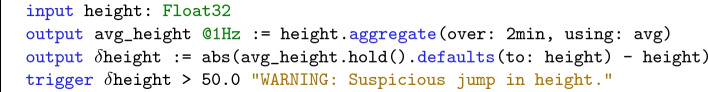


Note that this is just a brief introduction to RTLola and the StreamLAB framework. For more details, the authors refer to [8, 11, 12, 22].

### FPGA as Monitoring Platform

An RTLola specification can be compiled into the hardware description language VHDL and subsequently realized on an FPGA as proposed by Baumeister et al. [8]. An FPGA as target platform for the monitor has several advantages in terms of improving the development process, reducing its cost, and increasing the overall confidence in the execution.

Since the FPGA is a separate module and thus decoupled from the control software, these components do not share processor time or memory. This especially means that control and monitoring computations happen in parallel. Further, the monitor itself parallelizes the computation of independent RTLola output streams with almost no additional overhead. This significantly accelerates the monitoring process [8]. The compiled VHDL specification allows for extensive static analyses. Most notably, the results include whether the board is sufficiently large in terms of look-up tables and storage capabilities to host the monitor, and the power consumption when idle or at peak performance. Lastly, an FPGA is the sweet spot between generality and specificity: it runs faster, is lighter, and consumes less energy than general purpose hardware while retaining a similar time-to-deployment. The latter combined with a drastically lower cost renders the FPGA superior to application-specific integrated circuits (ASIC) during development phase. After that, when the specification is fixed, an ASIC might be considered for its yet increased performance.

### RTLola Specifications

**Fig. 5.**
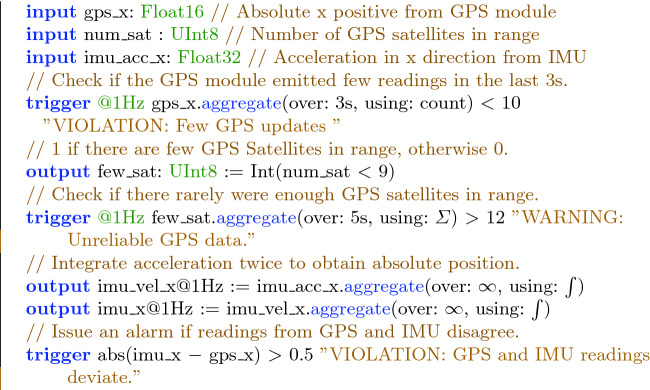
An RTLola specification validating GPS sensor data and cross validating readings from the GPS module and IMU.

The entire specification for the mission is comprised of three sub-specifications. This section briefly outlines each of them and explains representative properties in Fig. 5. The complete specifications as well as a detailed description were presented in earlier work [6, 21] and the technical report of this paper [7].

- **Sensor Validation.** Sensors can produce incorrect values, e.g. when too few GPS satellites are in range for an accurate trilateration or if the aircraft flies above the range of a radio altimeter. A simple exemplary validation is to check whether the measured altitude is non-negative. If such a check fails, the values are meaningless, so the system should not take them into account in its computations.
- **Geo-Fence.** During the mission, the aircraft has permission to fly inside a zone delimited by a polygon, called a geo-fence. The specification checks whether a face of the fence has been crossed, in which case the aircraft needs to ensure that it does not stray further from the permitted zone.
- **Sensor Cross-Validation.** Sensor redundancy allows for validating a sensor reading by comparing it against readings of other sensors. An agreement between the values raises the confidence in their correctness. An example is the cross-validation of the GPS module against the accelerometer. Integrating the readings of the latter twice yields an absolute position which can be compared against the GPS position.

Figure 5 points out some representative sub-properties of the previously described specification in RTLola, which are too long to discuss them in detail. It contains a validation of GPS readings as well as a cross-validation of the GPS module against the Inertial Measurement Unit (IMU). The specification declares three input streams, the *x*-position and number of GPS satellites in range from the GPS module, and the acceleration in *x*-direction according to the IMU.

The first trigger counts the number of updates received from the GPS module by counting how often the input stream  gets updated to validate the timing behavior of the module.

The output stream  computes the indicator function for  , which indicates that the GPS module might report unreliable data due to few satellites in reach. If this happens more than 12 times within five seconds, the next trigger issues a warning to indicate that the incoming GPS values might be inaccurate. The last trigger checks whether the double integral of the IMU acceleration coincides with the GPS position up to a threshold of 0.5 m.

### VHDL Synthesis

The specifications mentioned above were compiled into VHDL and realized on the Xilinx ZC702 Base Board^4^. The following table details the resource consumption of each sub-specification reported by the synthesis tool Vivado.

**Table:** 

Spec	FF	FF[$\%$]	LUT	LUT[$\%$]	MUX	Idle [mW]	Peak [W]
Geo-fence	2,853	3	26,181	71	4	149	1.871
Validation	4,792	5	34,630	67	104	156	2.085
Cross	3,441	4	23,261	46	99	150	1.911

The number of flip-flops (FF) indicates the memory consumption in bits; neither specification requires more than 600B of memory. The number of LUTs (Look-up Tables) is an indicator for the complexity of the logic. The sensor validation, despite being significantly longer than the cross-validation, requires the least amount of LUTs. The reason is that its computations are simple in comparison: Rather than computing sliding window aggregations or line intersections, it mainly consists of simple thresholding. The number of multiplexers (MUX) reflects this as well: Since thresholding requires comparisons, which translate to multiplexers, the validation requires twice as many of them. Lastly, the power consumption of the monitor is extremely low: When idle, neither specification requires more than 156mW and even under peak pressure, the power consumption does not exceed 2.1W. For comparison, a Raspberry Pi needs between 1.1W (Model 2B) and 2.7W (Model 4B) when idle and roughly twice as much under peak pressure, i.e., 2.1W and 6.4W, respectively.^5^

**Fig. 6.**
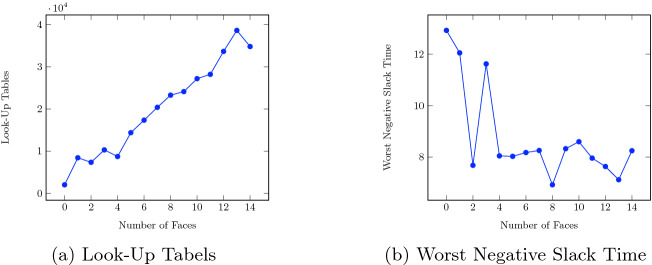
Result of the static analysis for different amounts of face of the geo-fence.

Note that the geo-fence specification checks for 12 intersections in parallel, one for each face of the fence (cf. Fig. 2). Adapting the number of faces allows for scaling the amount of FPGA resources required, as can be seen in Fig. 6a. The graph does not grow linearly because the realization problem of VHDL code onto an FPGA is a multi-dimensional optimization problem with several pareto-optimal solutions. Under default settings, the optimizer found a solution for four faces that required fewer LUTs than for three faces. At the same time, the worst negative slack time (WNST) of the four-face solution was lower than the WNST for the three-face solution as well (cf. Fig. 6b), indicating that the former performs worst in terms of running time.

## Results

As the title of the paper suggests, the superARTIS with the RTLola monitor component is cleared to fly and a flight test is already scheduled. In the meantime, the monitor was validated on log files from past missions of the superARTIS replayed under realistic conditions. During a flight, the controller polls samples from sensors, estimates the current position, and sends the respective data to the logger and monitor. In the replay setting, the process remains the same except for one detail: Rather than receiving data from the actual sensors, the data sent to the controller is read from a past log file in the same frequency in which they were recorded. The timing and logging behavior is equivalent to a real execution. This especially means that the replayed data points will be recorded again in the same way. Control computations take place on a machine identical to the one on the actual aircraft. As a result, from the point of view of the monitor, the replay mode and the actual flight are indistinguishable. Note that the setup is open-loop, i.e. , the monitor cannot influence the running system. Therefore, the replay mode using real data is more realistic than a high-fidelity simulation.

When monitoring the geo-fence of the reconnaissance mission in Fig. 2, all twelve face crossings were detected successfully. Additionally, when replaying the sensor data of the experiment in the enclosed backyard from Sect. 2.1, the erratic GPS sensor data lead to 113 violations regarding the GPS module on its own. Note that many of these violations point to the same culprit: a low number of available GPS satellites, for example, correlates with the occurrence of peaks in the GPS velocity. Moreover, the cross validation issued another 36 alarms due to a divergence of IMU and GPS readings. Other checks, for example detecting a deterioration of the GPS module based on its output frequency, were not violated in either flight and thus not reported.

## Conclusion

We have presented the integration of a hardware-based monitor into the superARTIS UAV. The distinguishing features of our approach are the high level of expressiveness of the RTLola specification language combined with the formal guarantees on the resource usage. The comprehensive tool framework facilitates the development of complex specifications, which can be validated on log data before they get translated into a hardware-based monitor. The automatic analysis of the specification derives the minimal requirements on the development board needed for safe operation. If they are met, the specification is realized on an FPGA and integrated into the superARTIS architecture. Our experience shows that the overall system works correctly and reliably, even without thorough system-level testing. This is due to the non-interfering instrumentation, the validated specification, and the formal guarantees on the absence of dynamic failures of the monitor.

## Footnotes

1 GPS modules only provide absolute position information; the first derivative thereof, however, is the velocity.

2 www.stream-lab.eu.

3 Details on how such a computation can cope with a statically-bounded amount of memory can be found in [12, 22].

4 https://www.xilinx.com/support/documentation/boards_and_kits/zc702_zvik/ug850-zc702-eval-bd.pdf.

5 Information collected from https://www.pidramble.com/wiki/benchmarks/power-consumption in January, 2020.
